# Early Detection of Inflammation-Prone STEMI Patients Using the CRP Troponin Test (CTT)

**DOI:** 10.3390/jcm11092453

**Published:** 2022-04-27

**Authors:** Rafael Y. Brzezinski, Ariel Melloul, Shlomo Berliner, Ilana Goldiner, Moshe Stark, Ori Rogowski, Shmuel Banai, Shani Shenhar-Tsarfaty, Yacov Shacham

**Affiliations:** 1 Internal Medicine “C”, “D”, and “E”, Tel Aviv Medical Center, Affiliated with the Sackler Faculty of Medicine, Tel Aviv University, Tel Aviv 69978, Israel; brzezinski@mail.tau.ac.il (R.Y.B.); arielme@tlvmc.gov.il (A.M.); berliners@tlvmc.gov.il (S.B.); orir@tlvmc.gov.il (O.R.); shanis@tlvmc.gov.il (S.S.-T.); 2 Department of Clinical Laboratories, Tel Aviv Medical Center, Affiliated with the Sackler Faculty of Medicine, Tel Aviv University, Tel Aviv 69978, Israel; ilanag@tlvmc.gov.il (I.G.); moshes@tlvmc.gov.il (M.S.); 3 Department of Cardiology, Tel Aviv Medical Center, Affiliated with the Sackler Faculty of Medicine, Tel Aviv University, Tel Aviv 69978, Israel; shmuelb@tlvmc.gov.il

**Keywords:** CRP, troponin, STEMI, acute coronary syndrome, inflammation

## Abstract

Elevated concentrations of C-reactive protein (CRP) early during an acute coronary syndrome (ACS) may reflect the magnitude of the inflammatory response to myocardial damage and are associated with worse outcome. However, the routine measurement of both CRP and cardiac troponin simultaneously in the setting of ST-segment myocardial infarction (STEMI) is not used broadly. Here, we sought to identify and characterize individuals who are prone to an elevated inflammatory response following STEMI by using a combined CRP and troponin test (CTT) and determine their short- and long-term outcome. We retrospectively examined 1186 patients with the diagnosis of acute STEMI, who had at least two successive measurements of combined CRP and cardiac troponin (up to 6 h apart), all within the first 48 h of admission. We used Chi-Square Automatic Interaction Detector (CHAID) tree analysis to determine which parameters, timing (baseline vs. serial measurements), and cut-offs should be used to predict mortality. Patients with high CRP concentrations (above 90th percentile, >33 mg/L) had higher 30 day and all-cause mortality rates compared to the rest of the cohort, regardless of their troponin test status (above or below 118,000 ng/L); 14.4% vs. 2.7%, *p* < 0.01. Furthermore, patients with both high CRP and high troponin levels on their second measurement had the highest 30-day mortality rates compared to the rest of the cohort; 21.4% vs. 3.7%, *p* < 0.01. These patients also had the highest all-cause mortality rates after a median follow-up of 4.5 years compared to the rest of the cohort; 42.9% vs. 12.7%, *p* < 0.01. In conclusion, serial measurements of both CRP and cardiac troponin might detect patients at increased risk for short-and long-term mortality following STEMI. We suggest the future use of the combined CTT as a potential early marker for inflammatory-prone patients with worse outcomes following ACS. This sub-type of patients might benefit from early anti-inflammatory therapy such as colchicine and anti-interleukin-1ß agents.

## 1. Introduction

Cardiac troponin is a sensitive and specific marker for myocardial injury, and its prognostic value in acute coronary syndrome (ACS) is well established [1]. Elevated concentrations of C-reactive protein (CRP) in patients with coronary artery disease (CAD) are associated with atherosclerotic disease activity and worse clinical outcomes [2,3,4,5]. Moreover, the rate of increase in CRP levels early after ACS onset is correlated with increased mortality rates and myocardial damage [6,7,8]. Finally, multivariable risk models have identified cardiac troponin and CRP as significant and independent predictors of risk in patients with ACS, with additive predictive value [5]. Yet, the routine measurement of both CRP and cardiac troponin simultaneously in the setting of ST-segment myocardial infarction (STEMI) is not used broadly and is still not recommended by recent clinical guidelines [9].

Systemic vascular inflammation plays a pivotal role in the progression of CAD, and several anti-inflammatory therapies have been examined in recent trials, including methotrexate [10], anti-interleukin (IL)-1ß [11], and colchicine [12], for the secondary prevention of atherosclerotic cardiovascular disease. However, it seems increased patient variability exists in the extent of the inflammatory response to cardiomyocyte necrosis and the progression of ACS [5]. Thus, more individualized approaches based on circulating inflammatory biomarkers are needed to identify patients who will benefit from these therapies.

Here, we sought to identify individuals who are prone to an elevated inflammatory response following STEMI using serial simultaneous measurements of both CRP and cardiac troponin-I during the first 48 h of admission. We characterized patients based on their CRP-Troponin-Test (CTT) results and determined their short- and long-term outcome.

## 2. Materials and Methods

### 2.1. Study Design and Clinical Data

We performed a retrospective, single-center observational study at the Tel-Aviv Sourasky Medical Center. We included consecutive patients admitted between January 2008 and January 2020 to the Cardiac Intensive Care Unit (CICU) with the diagnosis of acute STEMI, who had at least two successive measurements of both CRP and cardiac troponin levels within the first 48 h of admission [6,8]. The CTT result was derived from test findings of a CRP and a troponin measurement carried out up to 6 h apart.

The diagnosis of STEMI was based on a typical history of chest pain, diagnostic electrocardiographic changes, and serial elevation of serum cardiac biomarkers [1]. Primary percutaneous coronary intervention (PCI) was performed in patients with symptoms ≤ 12 h in duration as well as in patients with symptoms lasting 12–24 h if the symptoms continued to persist during hospitalization.

CAD was defined if a ≥50% narrowing in an epicardial coronary artery was present, or a history of coronary intervention (stent or angioplasty) to an epicardial coronary artery, or bypass surgery. CAD severity was divided into 4 categories according to the number of diseased vessels (i.e., 0, 1, 2, or 3) as previously described [13,14]. Following primary PCI left ventricular (LV) ejection fraction was measured in all patients by bedside echocardiography, within the first 48 h of admission.

The MDClone platform was used to automatically extract multiple demographic and clinical variables from Electronic Health Records as well as determine 30-day and all-cause mortality rates [15]. Missing data on cardiovascular history, clinical risk factors, treatment characteristics, and laboratory results were manually retrieved from the patients’ medical files. The median follow-up time for all-cause mortality was 4.5 years (interquartile range [IQR] 3.1–6.3).

### 2.2. Laboratory Tests

Complete blood count parameters were measured with a Coulter STKS electronic counter. Blood samples for CRP and cardiac troponin assessments were drawn in all patients upon admission to the emergency department or at the catheterization laboratory prior to primary PCI. A second sample was drawn following primary PCI, and within 48 h from CICU admission.

The white blood count (WBC) was determined by the Coulter STKS (Beckman Coulter, Nyon, Switzerland) electronic cell analyzer. Wide range C-reactive protein (CRP) levels were determined by the Bayer wr-CRP assay (Bayer, Leverkusen, Germany) [16]. High-sensitivity cardiac troponin I was measured by an ADVIA Centaur^®^ TnI-Ultra^®^ assay (Siemens, Munich, Germany).

### 2.3. Statistical Analysis

All continuous variables are displayed as means (±standard deviation (SD)) for normally distributed variables or median (interquartile range (IQR)) for variables with abnormal distribution. Categorical variables are displayed as numbers (%) of subjects within each group. Continuous variables were compared by a student’s *t*-test for normally distributed variables and by the Mann–Whitney U test for non-normally distributed ones. To assess associations among categorical variables, we used a Chi-square test. We assessed normal distributions using Kolmogorov–Smirnov’s test and Q-Q plots. The correlations between continuous variables were assessed by Pearson’s r for normally distributed variables and Spearmen’s r for variables with abnormal distribution.

To determine which CRP and troponin measurements (baseline vs. serial measurements), and corresponding cut-offs should be used to predict 30-day mortality, we used Chi-square automatic interaction detection (CHAID) [17]. CHAID analysis builds a predictive model to determine the best cutoffs for the input variables to predict an outcome. In CHAID, continuous predictors are split into categories with an approximately equal number of observations. CHAID creates all possible cross-tabulations for each categorical predictor until the best outcome is achieved and no further splitting can be performed. Patients were divided into groups according to the output cutoffs.

To adjust for possible confounding variables, we used a binary logistic regression to predict 30-day mortality status. We adjusted our model for the combined second CTT test status, age, sex, and conventional risk factors including history of heart failure, prior myocardial infarction (MI), diabetes, hypertension, and hyperlipidemia. Adjusted odds ratios (OR) with 95% confidence intervals (CI) were reported.

A two-tailed *p* < 0.05 was considered statistically significant. All analyses were performed with the SPSS (IBM SPSS Statistics, version 28, IBM Corp., Armonk, NY, USA, 2016) and GraphPad Prism version 9.00 (GraphPad Software, La Jolla, CA, USA).

## 3. Results

### 3.1. Patient Characteristics

**Figure 1:**
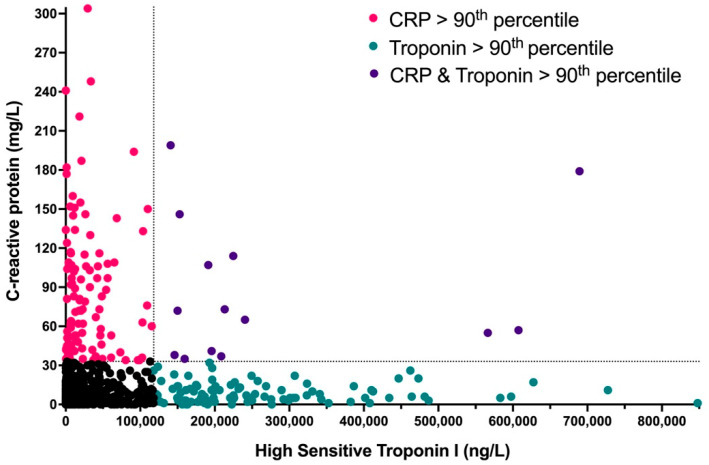
**Combined CRP and Troponin Test (CTT) results.** A scatterplot of the study population according to their second CRP and cardiac troponin test results. The dotted lines represent the 90th percentile thresholds (CRP > 33 mg/L and cardiac troponin > 118,000 ng/L) according to our CHIAD analysis as described in the Methods section.

Patient characteristics are displayed in Table 1. The final study cohort included 1186 STEMI patients. The mean age was 63.2 ± 13 years, and 207 patients (17.5%) were women. The distribution of the second CTT test results is presented in Figure 1.

The median first and second CRP levels were 4.3 [IQR 1.5–11.1] and 5 [IQR 1.6–11.8] mg/L, respectively. The corresponding median first and second troponin levels were 430.5 [64.7–4967.5] and 9995.5 [1778.7–43,631.7] ng/L. The correlation between CRP and cardiac troponin was r = 0.28 for the first measurement, and r = 0.22 for the second measurement, *p* < 0.01 for both. Notably, the correlation between the first troponin and the second CRP was stronger (r = 0.37, *p* < 0.01) and might reflect the slower rate of CRP increase compared to cardiac troponin following myocardial infarction [18].

A total of 46 patients (3.9%) died within 30 days of admission (mean age = 80.3 ± 10 years). We used CHAID tree analysis to determine which CTT measurement and cutoffs should be used to predict 30-day mortality. A second CRP level > 33 mg/ L (90th percentile of the cohort) was associated with the highest 30-day mortality rate. Accordingly, patients were divided into four groups based on their combined CTT results, i.e., CRP/troponin levels above or below the 90th percentile (Figure 1 and Table 1).

Patients with both high CRP (>33 mg/L) and high troponin (>118,000 ng/L) levels were older and had a higher prevalence of diabetes mellitus and prior heart failure compared to the rest of the cohort (Table 1). Individuals with high CRP levels had a higher prevalence of hypertension, regardless of their troponin test result (Table 1). Of note, the severity of CAD was similar across all groups.

### 3.2. CTT Results and 30-Day/All-Cause Mortality

**Figure 2:**
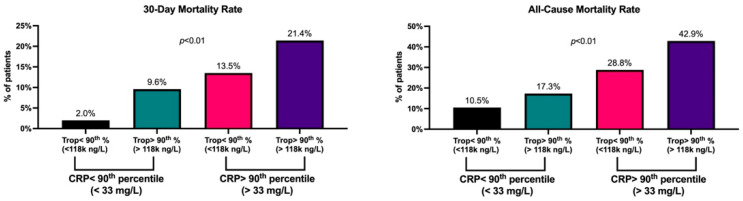
**Combined CRP and Troponin Test (CTT) results and mortality rates.** Bar graphs representing the proportion of patients who died during follow-up according to their CRP Troponin Test (CTT) results. The median follow-up time for all-cause mortality was 4.5 years [IQR 3.1–6.3]. The bar colors correspond to the 4 groups presented in Figure 1. *p*-values were calculated by the Chi-Square test.

Patients with high CRP concentrations (above the 90th percentile, >33mg/L) had higher 30-day and all-cause mortality rates regardless of their troponin test status (above or below 118,000 ng/L); 14.4% vs. 2.7%, *p* < 0.01 (Figure 2). However, patients with both high CRP and high troponin levels had the highest mortality rates compared to the rest of the cohort; 21.4% vs. 3.7%, *p* < 0.01 (Figure 2). We also assessed all-cause mortality after a median follow-up of 4.5 years [IQR 3.1–6.3] and observed a similar trend across all CTT test result categories (Figure 2). Patients with both high CRP and high troponin levels on their second measurement had the highest mortality rates compared to the rest of the cohort; 42.9% vs. 12.7%, *p* < 0.01 (Figure 2).

Finally, a binary logistic regression analysis showed that the combined result of the second CTT significantly predicted 30-day mortality after adjusting for age, sex, and conventional risk factors; OR = 6.98 95% CI 1.4, 35.5, *p* = 0.02 for patients with both high CRP and high troponin on their second measurement (above 90th percentile). Age and a history of heart failure were also significant predictors in the model (Table 2).

## 4. Discussion

The main findings of our study are that serial simultaneous measurements of cardiac troponin and CRP within the first 48 h of admission might identify patients at increased risk for short- and long-term mortality following STEMI. Patients with both high CRP and high troponin levels (above 90th percentile) were at increased risk for 30-day and all-cause mortality during a median follow-up of 4.5 years. We propose here the use of the combined CTT as an early and simple marker for inflammatory-prone patients with worse outcomes following ACS.

Our results portray the wide spectrum of the human inflammatory response to MI by considering cardiac troponin as the “antigen” in this relatively sterile inflammatory process. We show that some individuals demonstrate low systemic inflammation (evidenced by low CRP levels) even in the presence of extremely high myocardial necrosis (cardiac troponin > 118,000 ng/L) (Figure 1). On the other hand, some individuals with very low levels of troponin had extremely high CRP levels (>33 mg/L) and appear to be more “inflammatory prone” to myocardial necrosis. These inflammatory-prone patients had increased 30-day and long-term mortality rates. Moreover, our findings show that patients with elevated levels of CRP during STEMI have higher mortality rates regardless of their troponin levels on presentation (Figure 2). These results are in line with several past reports on the prognostic value of CRP in ACS [2,19,20,21,22,23,24]. Our findings are also in line with existing reports on the association between CRP levels and infarct size in STEMI patients, as well as the development of cardiac remodeling and microvascular disease during follow-up [25,26,27,28,29]. The cutoffs for “high” CRP in these reports ranged from 20–40 mg/L and are relatively similar to the ones used in our analysis. The higher mortality rates of patients with both high CRP and high troponin could be explained by the higher prevalence of heart failure and diabetes among these individuals. Nonetheless, it is reasonable to speculate that their inflammatory-prone nature played a major part in the development of these existing comorbidities in the first place, and thereby underline the importance of our findings.

Recent studies have suggested that serial CRP measurements can help reclassify stable and unstable CAD patients undergoing coronary interventions by assessing their “residual inflammatory risk” in terms of long-term outcomes [5,21,30,31]. The time interval between CRP measurements in these reports was around 4 weeks. Others have suggested a combined approach of multiple inflammatory biomarkers for improved risk stratification during STEMI [23,24,27,32]. Moreover, we and others have demonstrated that CRP velocity between two serial measurements within the first days of hospitalization is associated with increased mortality, left ventricular dysfunction, microvascular damage, and acute kidney injury [6,7,33,34]. We suggest here the addition of high sensitivity cardiac troponin to CRP across all time points to further improve risk stratification of CAD patients that underwent a coronary intervention. Most importantly, the CTT could be used to identify the precise subtype of patients that might benefit from early anti-inflammatory therapy, such as colchicine [12] and anti-IL-1ß agents [30]. We hypothesize that our “inflammatory-prone” patients who had high CRP levels—even in the presence of relatively low or normal troponin levels—are the ones who are most likely to benefit from these treatments.

Our study has several limitations. First, the retrospective nature of this study and the non-standardized timing of both CRP and troponin measurements poses a risk of residual confounding. Furthermore, the maximum time interval between the first and second CTTs was arbitrarily set at 48 h from admission. Future standardized, large-scale efforts are needed to determine the optimal time intervals between serial measurements. Finally, the cutoffs for both CRP and troponin levels used here were optimized for this specific study sample and may not present the same diagnostic yield in other populations. Moreover, the relatively small group of patients with both high CRP and troponin (*n* = 14) limits the statistical power of additional analyses aimed to detect residual confounding. Future studies should evaluate these cutoffs in other non-Caucasian populations and also explore the possibility of sex-specific thresholds [35]. Furthermore, large-scale studies should stratify patients according to pre-existing comorbidities, especially heart failure, in their analysis to possibly define specific thresholds for these patients.

In summary, we suggest further investigation of the novel CTT as an early clinical assessment tool for identifying patients at increased risk for worse outcome following STEMI. Future clinical trials should investigate the usefulness of the CTT in improving optimal patient selection to receive novel anti-inflammatory agents in the setting of ACS.

## Figures and Tables

**Table 1:** Baseline clinical characteristics by CTT * results (total *n* = 1186).

	CRP < 90th Percentile (<33 mg/L)		CRP > 90th Percentile (>33 mg/L)		*p*-Value
	Trop < 90th %ile (<118,000 ng/L)	Trop > 90th %ile (>118,000 ng/L)	Trop < 90th %ile (<118,000 ng/L)	Trop > 90th %ile (>118,000 ng/L)	
*n*	964	104	104	14	
Age, years (±SD)	62.6 (12.9)	62.9 (13.9)	67.9 (14.5)	68.0 (15.4)	<0.01
Women, *n* (%)	176 (18)	8 (8)	22 (21)	1 (7)	0.03
Ejection fraction, % (±SD)	38.3 (19.6)	32.9 (16.6)	33.8 (19.6)	27.9 (16.5)	<0.01
Diabetes, *n* (%)	243 (25)	18 (17)	43 (41)	7 (50)	<0.01
History of HF, *n* (%)	95 (10)	22 (21)	25 (24)	8 (57)	<0.01
Past MI, *n* (%)	174 18.1)	19 (18.3)	28 (27)	1 (7.1)	0.11
Hyperlipidemia, *n* (%)	499 (52)	46 (44)	59 (57)	10 (71)	0.13
Current smoker, *n* (%)	466 (49)	55 (54)	44 (44)	3 (23)	0.13
Hypertension, *n* (%)	449 (47)	39 (38)	61 (59)	7 (50)	0.02
**CAD severity**					0.11
0 diseased vessels, *n* (%)	6 (1)	0 (0)	1 (1)	0 (0)	
1 diseased vessel, *n* (%)	378 (40)	46 (44)	32 (33)	2 (15)	
2 diseased vessels, *n* (%)	313 (33)	31 (30)	24 (25)	7 (54)	
3 diseased vessels, *n* (%)	255 (27)	27 (26)	39 (41)	4 (31)	
Glucose, mg/dL (±SD)	150.3 (60.8)	154.5 (67.0)	170.7 (89.5)	220.7 (109.3)	<0.01
HbA1C, % (±SD)	5.9 [5.6, 6.5]	5.8 [5.5, 6.3]	6.0 [5.7, 7.1]	5.8 [5.7, 6.5]	0.15
WBC, 10^9^/L (±SD)	11.8 (4.2)	13.4 (3.8)	12.6 (4.3)	14.9 (5.2)	<0.01
1st CRP, mg/L [IQR]	3.5 [1.3, 8.2]	4.2 [1.4, 7.8]	58.0 [43.1, 109.8]	66.6 [25.5, 119.9]	<0.01
2nd CRP, mg/L [IQR]	3.9 [1.4, 8.8]	5.8 [2.9, 12.6]	72.7 [45.3, 108.7]	68.8 [44.3, 112.3]	<0.01
1st Troponin, ng/L [IQR])	299.5 [48.0, 2618.8]	6021.5 [137.0, 54,233.2]	8127.5 [1770.0, 23,908.5]	33,484.0 [7278.2, 125,331.0]	<0.01
2nd Troponin, ng/L [IQR])	6689.0 [1192.0, 23,949.8]	222,112.0 [174,063.8, 307,510.2]	18,453.5 [6795.0, 42,399.5]	202,177.0 [154,482.0, 236,586.5]	<0.01

* CTT—CRP Troponin Test (second measurement); CRP—C-Reactive Protein; HbA1c—Hemoglobin A1c; HF—Heart Failure; MI—Myocardial Infarction; PLT—Platelets; Trop—Cardiac Troponin I; WBC—White Blood Count.

**Table 2:** Binary logistic regression to predict 30-day mortality.

	Odds Ratio	95.0% CI		*p* Value
		Lower	Upper	
Age (years)	1.092	1.053	1.132	<0.01
Sex (women)	1.825	0.787	4.233	0.16
Diabetes mellitus	1.993	0.935	4.246	0.07
History of HF	10.914	5.201	22.903	<0.01
Past MI	0.896	0.387	2.078	0.79
Hyperlipidemia	1.320	0.584	2.984	0.5
Hypertension	1.451	0.578	3.642	0.43
2nd CTT result (normal CRP and troponin as indicator)				
High troponin-normal CRP	6.896	2.531	18.788	<0.01
Normal troponin-high CRP	4.756	1.960	11.543	<0.01
High troponin-high CRP	6.974	1.372	35.457	0.02

Method = Enter. CTT—CRP Troponin Test (second measurement); CRP—C-Reactive Protein; HF—Heart Failure.

## Data Availability

Data will be made available upon reasonable request.
